# Diagnostic accuracy of calprotectin in periprosthetic joint infection: a diagnostic meta-analysis

**DOI:** 10.1186/s13018-021-02895-4

**Published:** 2022-01-06

**Authors:** Jisi Xing, Jiahao Li, Zijian Yan, Yijin Li, Xiaofang Liu, Lilei He, Ting Xu, Changbing Wang, Lilian Zhao, Ke Jie

**Affiliations:** 1 Foshan Hospital of Traditional Chinese Medicine Affiliated to Guangzhou University of Chinese Medicine, Foshan Hospital of Traditional Chinese Medicine, Foshan, 528000 Guangdong Province China; 2 The First Clinical Medical School, Guangzhou University of Chinese Medicine, Jichang Road 12#, District Baiyun, Guangzhou, Guangdong China; 3 Foshan Hospital of Traditional Chinese Medicine, Foshan, 528000 Guangdong Province China

**Keywords:** Calprotectin, Diagnosis, Periprosthetic joint infection, Meta-analysis

## Abstract

**Background:**

Periprosthetic joint infection (PJI) is considered to be one of the most challenging complications of joint replacement, which remains unpredictable. As a simple and emerging biomarker, calprotectin (CLP) has been considered to be useful in ruling out PJI in recent years. The purpose of this study was to investigate the accuracy and sensitivity of CLP in the diagnosis of PJI.

**Methods:**

We searched and screened the publications from PubMed, Web of Science, EMBASE, and Cochrane Library from database establishment to June 2021. Subsequently, Stata version 16.0 software was used to combine the pooled sensitivity, specificity, positive likelihood ratio (PLR), negative likelihood ratio (NLR), diagnostic odds ratio (DOR), operating characteristic curve, and area under the curve (AUC). Heterogeneity across articles was evaluated by the I^2^ statistics. Finally, sources of heterogeneity were detected by subgroup analysis based on study design, detection method, sample size, and cutoff values.

**Results:**

A total of 7 studies were included in our study, comprising 525 patients. The pooled sensitivity, specificity, PLR, and NLR of CLP for PJI diagnosis were 0.94(95% CI 0.87–0.98), 0.93(95% CI 0.87–0.96), 13.65(95% CI 6.89–27.08), and 0.06(95% CI 0.02–0.15), respectively, while the DOR and AUC were 222.33(95% CI 52.52–941.11) and 0.98 (95% CI 0.96–0.99), respectively.

**Conclusion:**

Synovial CLP is a reliable biomarker and can be used as a diagnostic criterion for PJI in the future. However, the uncertainty resulting from the poor study numbers and sample sizes limit our ability to definitely draw conclusions on the basis of our study.

**Supplementary Information:**

The online version contains supplementary material available at 10.1186/s13018-021-02895-4.

## Introduction

Periprosthetic joint infection (PJI) is one of the most serious complications after arthroplasty. Infections accounted for 25% of revisions, and its prevalence is expected to increase notably over the next decades [1]. PJI will not only aggravate the financial burden of patients, but also affect quality of life and interfere with joint function [2, 3]. Since the symptoms of PJI are usually non-specific, it is difficult to diagnose PJI accurately and quickly, which delays the optimal treatment time for PJI and prevents patients from achieving a satisfactory prognosis [4]. Consequently, various diagnostic guidelines and criteria have been proposed, including the International Consensus Meeting (ICM) diagnostic criteria, American Academy of Orthopedic Surgeon (AAOS)’s guidelines, and Musculoskeletal Infection Society (MSIS) [5–7]. Although there are currently a series of diagnostic guidelines, PJI may be present without meeting these algorithms clinically, specifically in patients with less virulent organisms and negative culture [8].

Therefore, there is a clinical need for a reliable and easily available diagnostic biomarker to achieve rapid diagnosis and differentiation in various situations. Calprotectin (CLP) is derived from neutrophils and macrophages. As an inflammatory reactant, its release and expression levels will increase in infection, trauma, and inflammatory diseases [9–11]. Follow-up studies focusing on the diagnostic accuracy of novel biomarkers have proved that CLP is a useful biomarker, which has the characteristics of rapid evaluation, high sensitivity, and specificity [12–18]. Nevertheless, because of the small sample size and inconsistent results, their conclusions have not been recognized. Therefore, given the ambiguities and uncertainties in the evidence [14], we conducted a systematic review and meta-analysis of these literatures to study the diagnostic value of CLP in PJI.

## Method

This article is conducted as claimed by recommendations of the Cochrane and follows the Preferred Reporting Items for Systematic Reviews and Meta-Analyses (PRISMA) guidelines [19]. Ethical approval was not necessary for this article and the research protocol had not been registered, because this research only involves the review of published articles. The research protocol is decided by all authors.

### Search strategy

Two researchers systematically conducted electronic searches to identify all eligible articles in the following databases: PubMed, Cochrane Library, EMBASE, Web of Science database, while the searches were performed from the inception of each database through to June 2021. Medical Subject Heading (MeSH) terms and entry terms contained in the search strategy were as follows: “Prosthesis-Related Infections” OR “Prosthesis Related Infections” OR “Infections, Prosthesis-Related” OR “Prosthesis-Related Infection” OR “Peri-Prosthetic Joint Infection” OR “Periprosthetic Joint Infection” OR “Prosthetic joint infection” OR “PJI” represented disease, “Calprotectin” OR “Calgranulin” OR “Leukocyte L1 Antigen Complex” stranded for target index.

### Selection of study

Literature was included if it met the following inclusion criteria: (1) using CLP as an index for the diagnosis of PJI, (2) the integrated data (true positive, false negative, false positive, true negative) were provided directly or indirectly, (3) A definite gold standard is used in the research, such as MSIS or ICM. The exclusion criteria mainly include: (1) animal studies; (2) studies with incomplete data; (3) reviews, comments, and letters.

### Data extraction and quality assessment

Relevant information was independently recorded by two reviewers from all selected studies, and the extracted data are input into a sheet in Excel. Variables extracted were: (1) first author, the year in which the article was published, the country, the design type of the study, location of arthroplasty; (2) gender, average age of patients, the gold standard, the detection method, and the cutoff value of CLP; (3) sensitivity, specificity, positive likelihood ratio (LR), negative LR, diagnostic odds ratio (DOR), area under the curve (AUC). Then, two researchers used The Quality Assessment of Diagnostic Accuracy Studies (QUADAS-2) [20] in the Revman (version5.4) software to evaluate the quality of all the literature, which is composed of patient selection, index test, reference standard, and flow and timing. If there is any disagreement in this process, the third author is responsible for making the decision.

### Statistical analysis

All extracted data analysis and picture production are performed with the Stata16.0 software. Bivariate random effect model was selected to analyze the tp, fp, fn, and tn values of 2 × 2 table recorded in the sheet and to test the heterogeneity. The sensitivity, specificity, positive likelihood ratio (PLR), negative likelihood ratio (NLR), diagnostic score, and diagnostic odds ratio (DOR) were calculated after integration. In addition, by drawing the summary receiver operating characteristics (SROC) through the Midas command, the calculated area under the curve (AUC) discriminates the diagnostic ability of CLP.

After that, the *I*^2^ statistics were performed to assess the heterogeneity of the studies. Statistically, the bigger the *I*^2^, the bigger the heterogeneity. If the heterogeneity of the article is too large, we will identify the source of the heterogeneity through subgroup analysis. Preplanned subgroups were designed according to the type of cutoff values used in the study, study design, sample size, detection method, and sample size.

The Deeks’ funnel plot was applied to evaluate the publication bias, while the Fagan plot was used to clearly reflect the change of the diagnostic value of CLP on the incidence of PJI.

## Results

### Characteristics of the included studies

**Fig. 1:**
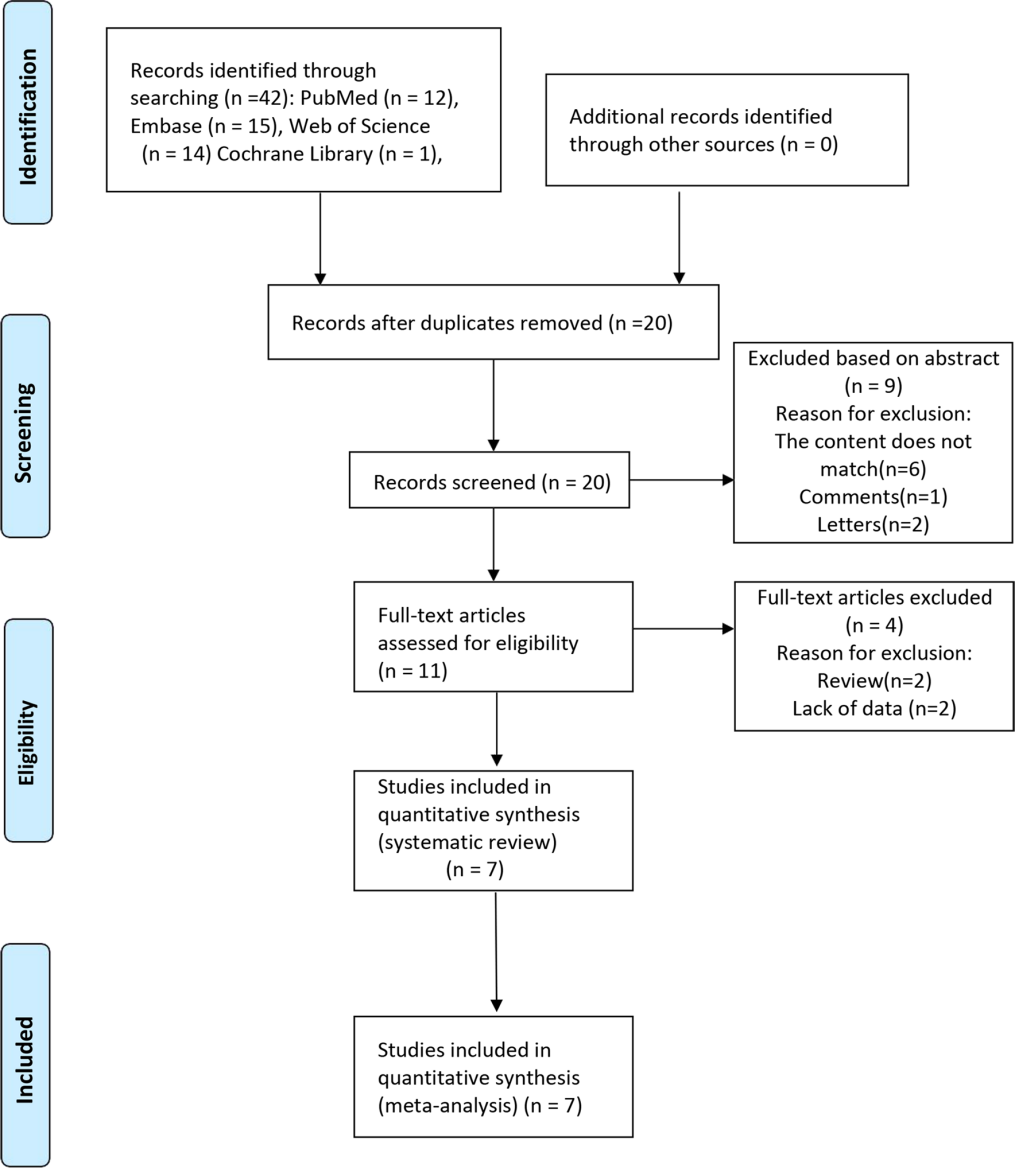
Flow diagram for study selection

The flowchart of the literature screening process is summarized in Fig. 1. An electronic search yielded 12 studies in PubMed (MEDLINE), 15 in EMBASE, 14 in Web of Science, and one in the Cochrane Library. No other publications were found by manual search. After the removal of 22 duplicates, 20 studies remained; Then 9 articles were excluded based on titles and abstracts, including 6 inconsistent contents, 2 letters, and 1 comment. Among the remaining 20 articles, 4 were deleted after reading the full article, including 2 literatures with insufficient data, and 2 reviews. This process ultimately resulted in 7 studies that were eligible for the final meta-analysis.

A total of 525 patients were enrolled in seven studies [12–18], including 320 patients with non-PJI and 205 patients with confirmed PJI. Most of these patients underwent knee or hip replacement, and some have undergone total joint replacements such as shoulder and elbow joints. Six of the studies were prospective and one was retrospective. In addition, the sample types used in the included articles were all synovial fluid, and one of them also measured calprotectin in blood. All studies provided methods for the detection of calprotectin. Three papers use ELISA and lateral flow assay, respectively, and one paper used both detection methods for comparison. Four studies regarded MSIS as the “gold standard” for the diagnosis of PJI, and three studies adopted ICM as “the gold standard” for diagnosis. Table 1 shows the baseline characteristics of all eligible studies. A summary of data extraction results (2 × 2 table) is offered in Table 2.

**Table 1:** Characteristics of the studies in meta-analysis for the diagnosis of PJI applying calprotectin

Study	Year	Country	Study design	Gender(M/F)	Median age*	Joint type	Detection method	Cutoff values	Gold standard
Bakker et al. [17]	2017	Netherlands	P	NA	NA	Hip/knee/shoulder	LFA	50 mg/L	MSIS
Bakker et al. [16]	2017	Netherlands	P	25/36	65/60	Hip/knee/shoulder/elbow	LFA	50 mg/L	MSIS
Salari et al. [13]	2019	Italy	P	36/40	69	Knee	ELISA	50 mg/L	ICM
Trotter et al. [14]	2020	UK	R	37/32	74.3	Hip/knee	LFA	50 mg/L	ICM/ICM-cr
Zhang et al. [18]	2020	China	P	21/42	64/57	Hip/knee	ELISA	173 ug/ml	MSIS
Grzelecki et al. [12]	2020	Poland	P	25/60	65.5/68.3	Hip/knee	ELISA	1.0 mg/L or 1.5 mg/L	ICM
Warren et al. [15]	2021	USA	P	57/66	66.9/65.4	Knee	ELISA/ LFA	50 mg/L	MSIS

*The values were given as the number with PJI/non-PJI

*P* prospective study, *R* retrospective study, *LFA* lateral flow assay, *NA* not applicable

**Table 2:** Data extracted for the construction of 2 × 2 table

Author	Year	TP	FP	FN	TN	Total
Bakker et al. a	2017	13	3	2	34	52
Bakker et al. b	2017	17	4	2	38	61
Salari et al	2019	28	2	0	42	72
Trotter et al	2020	18	11	6	34	69
Zhang et al	2020	20	1	1	41	63
Grzelecki et al	2020	43	2	2	38	85
Warren et al	2021	52	3	1	67	123

*TP* true positive, *FP* false positive, *FN* false negative, *TN* true negative

### Quality assessment and publication biases

**Fig. 2:**
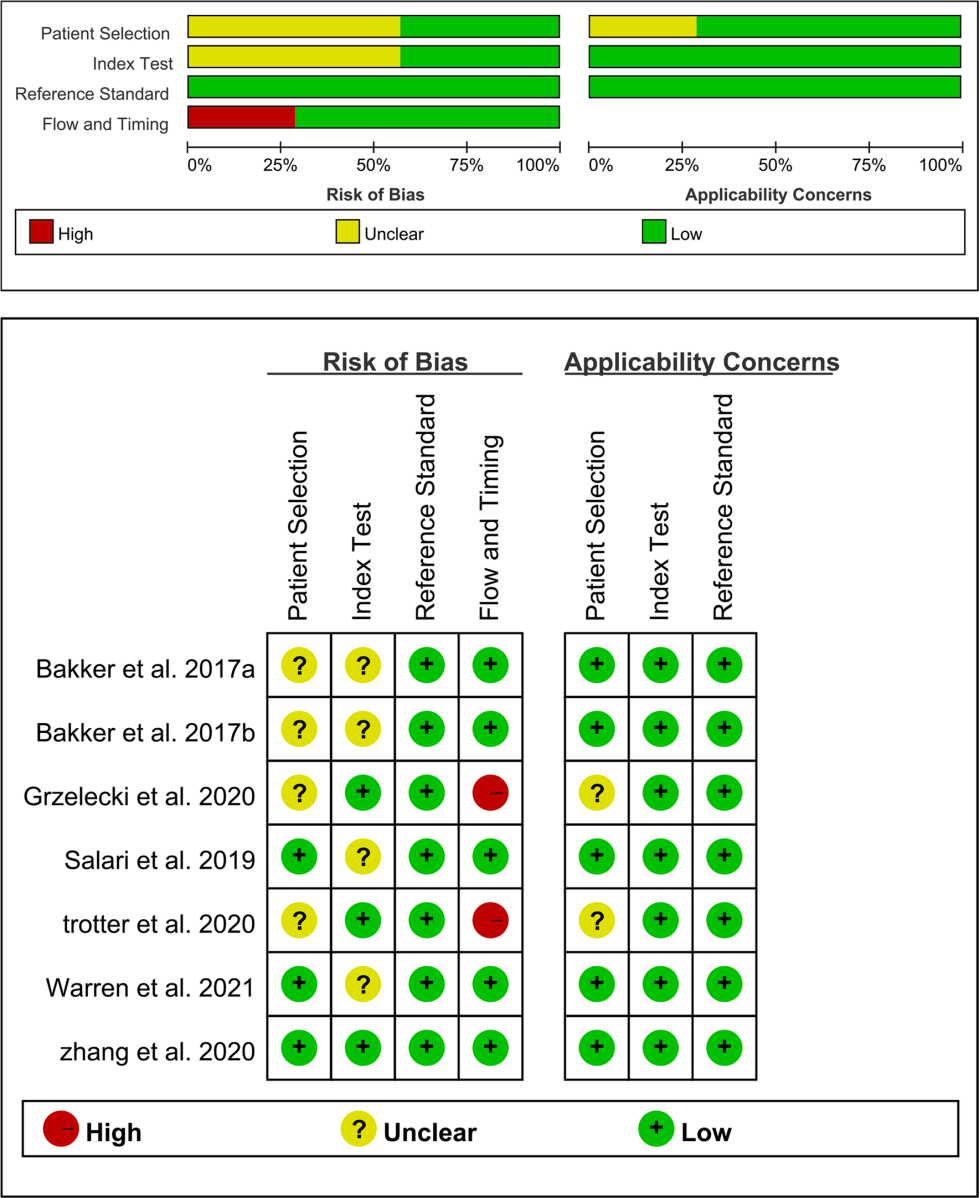
Quality assessment of included studies based on QUADAS-2 tool criteria

**Fig. 3:**
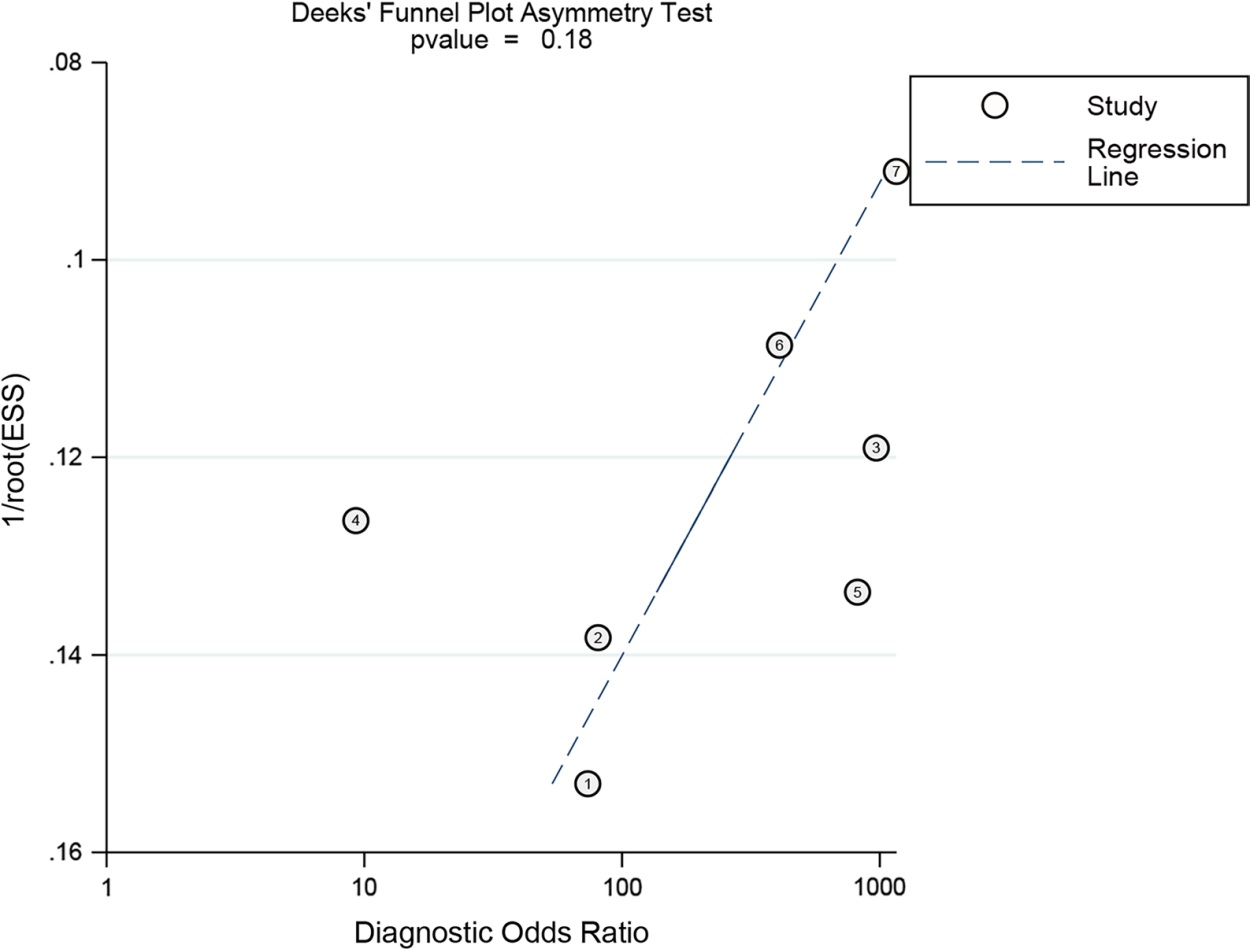
Funnel plot for publication bias assessment of included studies

The quality assessment results of 7 studies using the QUADAS-2 scale are indicated in Fig. 2. The figure shows that the overall quality of the included studies was good, with only two studies are “high risks,” and the rest are “unclear” or “low risk.” Although the sample size of the included literature is relatively small, the quality of the research is persuasive. In addition, the funnel plot asymmetry demonstrated no obvious publication bias was detected (*P* = 0.18) (Fig. 3).

### Diagnostic accuracy of calprotectin for PJI

**Fig. 4:**
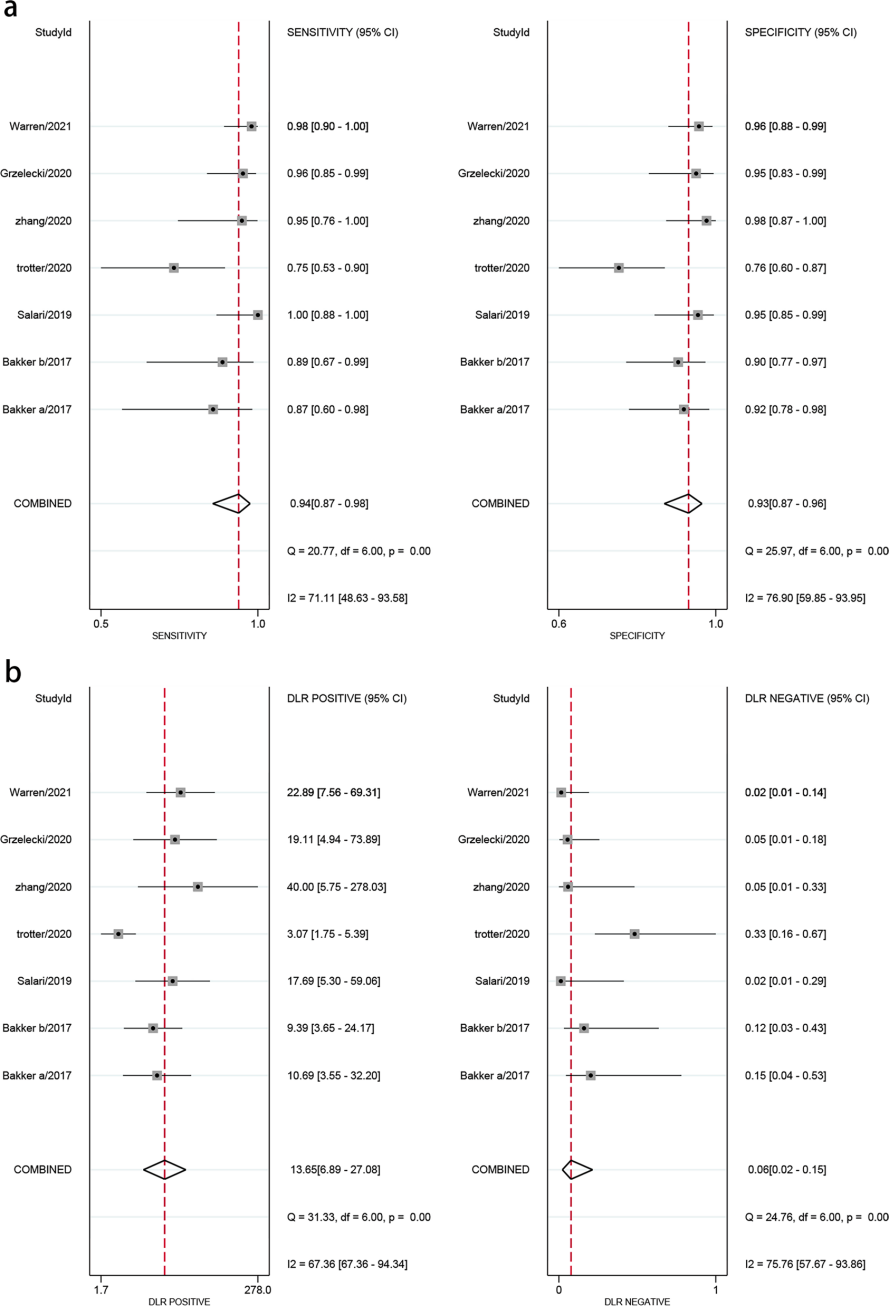
Forest plot of CLP for PJI. **a** Pooled sensitivity and specificity. **b** Pooled diagnostic score and diagnostic odds ratio

**Fig. 5:**
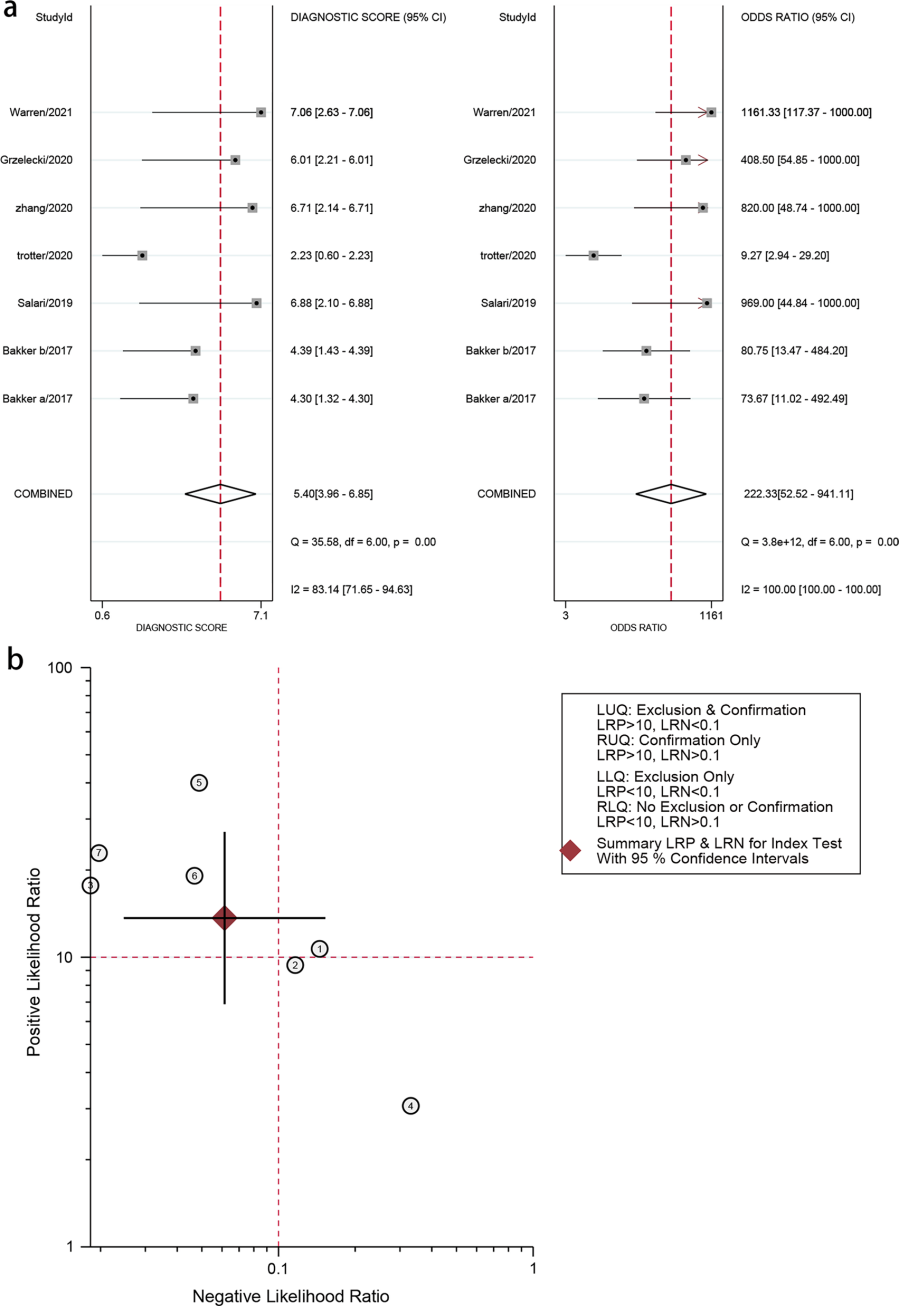
Forest plots of likelihood ratio (**a**) and likelihood ratio scatter diagrams (**b**)

**Fig. 6:**
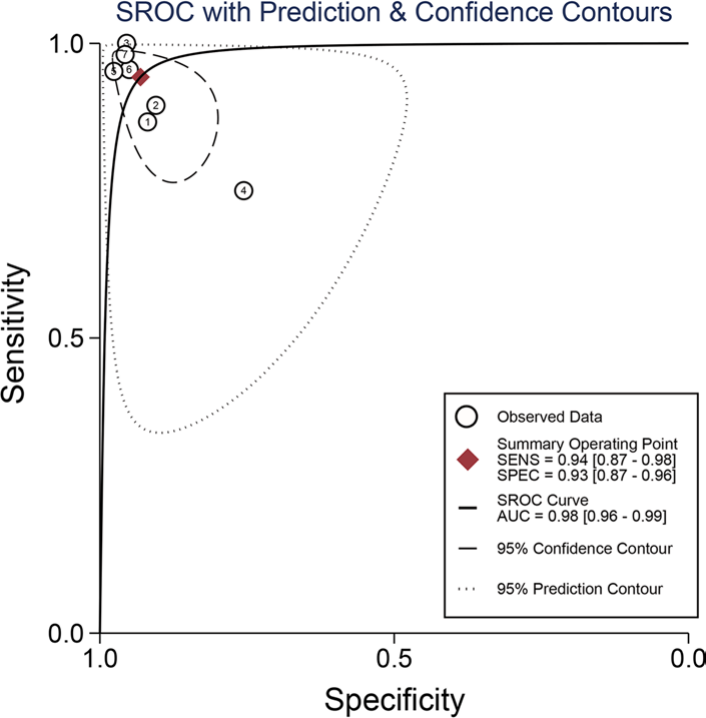
SROC curve of included studies

**Fig. 7:**
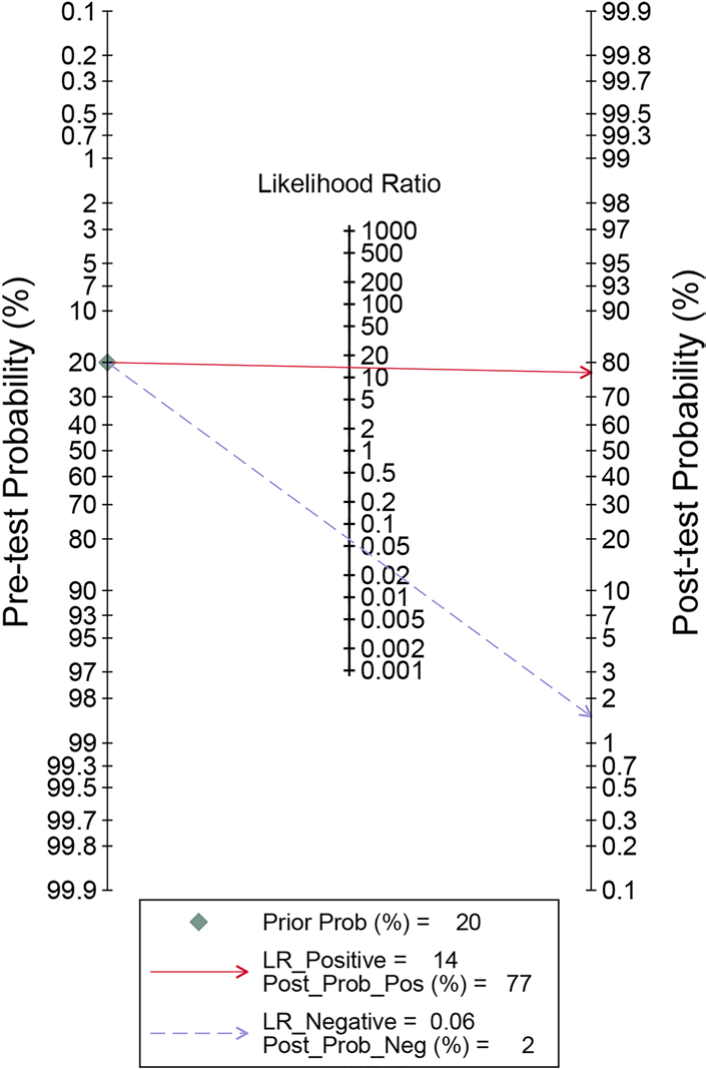
Fagan’s nomogram of the CLP for diagnosis of PJI

Forest plots in Fig. 4 revealed that the pooled sensitivity across studies for CLP was 0.94(95% CI 0.87–0.98), the pooled specificity was 0.93(95% CI 0.87–0.96), the pooled positive LR was 13.65(95% CI 6.89–27.08), the pooled negative LR was 0.06(95% CI 0.02–0.15), and the pooled DOR was 222.33(95% CI 52.52–941.11) (Fig. 5). The *I*^2^ statistics for sensitivity and specificity were 71.1% (95% CI 48.6–93.6) and 76.9% (95% CI 59.9–94.0), showing that there was significant heterogeneity. The SROC curve indicated the sensitivity and specificity, as well as the prediction regions, with an AUC of 0.98 (95% CI 0.96–0.99) (Fig. 6). As shown in Fig. 7, the Fagan plot demonstrated that a positive result on the CLP test increased the probability of PJI from 14 to 77% and a negative result on the CLP test decreased the probability of PJI to 2%.

### Subgroup analysis

For all 7 studies, the heterogeneity (*I*^2^) was shown for sensitivity and specificity among studies of both index tests. Thus, we performed a subgroup analysis of factors that may be the possible sources of heterogeneity, including study design, detection method, sample size, and cutoff values (Table 3). When only analyzing 6 prospective studies, the sensitivity and specificity of CLP increased to 0.96 (95% CI 0.91–0.98) and 0.95(95% CI 0.91–0.97), respectively, while I^2^ statistics decreased significantly (24.21% vs. 71.11%, 0.00% vs. 76.9%), suggesting that study design is the source of heterogeneity. In addition, PJI was more diagnostically accurate in studies using ELISA as the detection method compared with studies using lateral flow assay.

**Table 3:** Subgroup analysis of CLP for PJI diagnosis

Subgroup analyses	No. of studies	Sensitivity (95% CI)	*I*^2^ (95% CI)	Specificity (95%CI)	*I*^2^ (95% CI)	PLN (95% CI)	NLR (95% CI)	Diagnostic score (95% CI)	DOR (95% CI)	AUC (95% CI)
Overall studies	7	0.94(0.87–0.98)	71.11(48.63–93.58)	0.93(0.87–0.96)	76.9(59.85–93.95)	13.65(6.89–27.08)	0.06(0.02–0.15)	5.40(3.96–6.85)	222.33(52.52–941.11)	0.98(0.96–0.99)
Prospective	6	0.96 (0.91–0.98)	24.21(0.00–89.32)	0.95(0.91–0.97)	0.00(0.00–100.00)	17.56 (10.49–29.38)	0.05 (0.02,0.10)	5.94(4.95–6.93)	378.51(140.62–1018.79)	0.98(0.96–0.99)
Detection method										
Lateral flow assay	4	0.91(0.76–0.97)	71.26(41.15–100.00)	0.9(0.80–0.95)	77.92(55.97–99.88)	9.09(4.01–20.61)	0.10(0.04–0.30)	4.49(2.74–6.23)	88.72(15.44–509.81)	0.96(0.94–0.97)
ELISA	4	0.97(0.93–0.99)	0.00(0.00–10.00)	0.96(0.92–0.98)	0.00(0.00–10.00)	23.83(12.08–47.01)	0.03(0.01–0.07)	6.73(5.51–7.95)	840.12(248.09–2845.01)	0.99(0.98–1.00)
Sample size										
< 100	6	0.93(0.83–0.97)	66.87(38.12–95.63)	0.93(0.85–0.96)	77.50(59.56–95.44)	12.47(5.83–26.65)	0.08(0.03–0.19)	5.09(3.57–6.61)	162.99(35.64–745.50)	0.97(0.96–0.98)
Cutoff values										
50 mg/L	5	0.94(0.81–0.98)	76.58(55.75–97.41)	0.91(0.83–0.96)	78.20(59.12–97.28)	10.97(5.05–23.85)	0.07(0.02–0.23)	5.07(3.24–6.91)	159.59(25.54–997.40)	0.97(0.95–0.98)

*I*^*2*^ inconsistency index, *AUC* area under the curve of summary receiver operating characteristic curves, *CI* confidence interval, *PLR* positive likelihood ratio, *NLR* negative likelihood ratio, *DOR* diagnostic odds ratio

## Discussion

Although the incidence of PJI is about 1–2%, the economic burden it brings is heavy, early diagnosis of PJI is the key to the effective reduction of patient burden and successful management [21]. Therefore, the ability to distinguish between septic and aseptic failure of the prosthesis is crucial, because the treatment of PJI patients to eradicate infected microorganisms is much more complicated [22]. The diagnosis of PJI depends on the combination of serologic testing, synovial fluid aspiration, radiographic evaluation, microbiology, and histopathological examination in addition to clinical symptoms [23, 24].

Currently, the only serum biomarkers recommended by the Infectious Diseases Society of America (IDSA) for the diagnostic evaluation of PJI are serum erythrocyte sedimentation rate (ESR) and C-reactive protein (CRP), which are not specific [5]. Hence, researchers have become increasingly aware of the significance of investigating and developing the emerging diagnostic biomarkers for PJI. It is gratifying that more and more biomarkers for the diagnosis of PJI have been discovered, including α-defensin, leukocyte esterase [LE], interleukin [IL]-6, IL-8, IL-10, IL-1b, Procalcitonin and Tumor Necrosis Factor Alpha, and so on [25, 26].

Recently, several investigators considered CLP as a promising biomarker. During inflammation, CLP is actively released and exerts a key role by stimulating leukocyte recruitment and inducing cytokine secretion, so it can be used as a biomarker for diagnosis and follow-up and a predictor of response to inflammation-related diseases [10, 11]. In the previous literature, CLP had been reported to predict or evaluate the progress of inflammatory diseases such as inflammatory bowel disease (IBD) [27, 28], rheumatoid arthritis [29, 30], and spondyloarthritis [31]. However, it was not until 2017 that Bakker et al. [16] first reported the role of CLP in diagnosing PJI. Because of its good diagnostic accuracy, an increasing number of studies have attempted to investigate the function of CLP. To our knowledge, our study is the first meta-analysis evaluating the ability of CLP in the diagnosis of PJI after a literature review.

Our study revealed that CLP indicated a comparable, extremely high diagnostic value to identify PJI (with a pooled sensitivity and specificity of 0.94(95% CI 0.87–0.98) and 0.93(95% CI 0.87–0.96), respectively). The pooled positive LR was 13.65(95% CI 6.89–27.08) and the pooled negative LR was 0.06(95% CI 0.02–0.15), with an AUC of 0.98 (95% CI 0.96–0.99). This result is much better than commonly used biomarkers, which may provide a new alternative for the diagnosis of PJI. The effectiveness of clinical diagnostic indicators is usually evaluated by LR and DOR. In the guide, LR +  > 5, LR −  < 0.2 or DOR > 10 are considered to be good predictive values, and LR +  > 2, LR −  < 5 or DOR > 4 are considered to be possible predictive values [32, 33]. Therefore, CLP is a superior predictor for diagnosing PJI, no matter when LR or DOR is used as the reference parameter. Another parameter widely used in diagnostic tests is the posttest probability, which reflects the probability of a PJI patient when the test result is negative or positive. The Fagan diagram shows that the CLP's ability to distinguish PJI is excellent.

Apparently, there is a degree of heterogeneity in the studies we pooled. Therefore, we performed a reasonable subgroup analysis to find the source of heterogeneity. The results of the subgroup analyses suggested that the heterogeneity may be the result of differences in the type of study and the detection method, and the heterogeneity is greatly reduced after removing the retrospective study. The study of Trotter et al. [14] is the only retrospective study included in the literature, and its overall accuracy of to diagnose PJI was 75.36%, which is lower than the other 6 studies. The authors suggest that the use of frozen storage samples may lead to leukocyte lysis and increased calprotectin during freeze–thaw process, while relevant study was lacking recently.

According to the results of subgroup analysis, the significance across studies heterogeneity could be accounted by the different tests. The methods of CLP measurement in our included studies were lateral flow test (LFA)or ELISA, and the results of the subgroup analysis show that the diagnostic accuracy for PJI using lateral flow assay obtained a lower accuracy in our analysis. A recent study by Suen et al. [34] indicated similar performance differences between the Synovial α­defensin lateral flow test and the α­defensin ELISA method. However, LFA is now a reliable diagnostic tool in numerous fields where portable, simple, and particularly rapid on-site detection methods are needed [35].

There were some limitations in our research. First, this study only included 7 articles, so the sample size was relatively small, with only 205 cases in the PJI group and 320 cases in the non-PJI group. Second, there is currently no gold standard for testing PJI, and it is possible that some positive patients are still missed because the gold standard cannot be tested. Due to limited data, we were unable to conduct subgroup analysis to compare the diagnostic accuracy of synovial fluid and serum CLP. And so far, there is not enough literature to clarify whether the CLP method can improve the outcome of patients receiving antibiotics.

## Conclusions

The present study indicated that CLP detection of PJI has good diagnostic accuracy and specificity. Hence, it can be considered a promising biomarker for the diagnosis of PJI. However, the present data remain insufficient and further studies regarding the combination of CLP and other biomarkers for diagnosing PJI are warranted.

## Supplementary Information

**Additional file 1.** Detailed search strategy of Pubmed.

## Data Availability

The datasets used and/or analyzed during the current study are not publicly available due to feasibility but are available from the corresponding author on reasonable request.

## Abbreviations

PJI: Periprosthetic joint infection
CLP: Calprotectin
AAOS: The American Academy of Orthopedic Surgeon
MSIS: Musculoskeletal Infection Society
ICM: International Consensus Meeting
LR: Likelihood ratio
DOR: Diagnostic odds ratio
SROC: Summary receiver operating characteristic
AUC: Area under the curve
